# Nutritional status assessed by prognostic nutritional index and its association with lower extremity deep venous thrombosis: a retrospective study

**DOI:** 10.3389/fnut.2026.1794087

**Published:** 2026-05-20

**Authors:** Zhaopeng Wang, Yongbin Shen, Jiaqi Huang, Peng Zhao, Fubin Xu

**Affiliations:** 1 Medical Department, The Second Affiliated Hospital of Harbin Medical University, Harbin, Heilongjiang, China; 2 Department of Vascular Surgery, The Second Affiliated Hospital of Harbin Medical University, Harbin, Heilongjiang, China; 3 Medical Insurance Office, The Second Affiliated Hospital of Harbin Medical University, Harbin, Heilongjiang, China; 4 Department of Ultrasound, The Second Affiliated Hospital of Harbin Medical University, Harbin, Heilongjiang, China

**Keywords:** deep venous thrombosis, hospitalised patients, LEDVT, nutritional status, prognostic nutritional index, risk prediction

## Abstract

**Background:**

Deep venous thrombosis (DVT) is a frequent and potentially fatal complication of hospitalised patients in the lower extremities. Nutritional status affects immune function, coagulation, and recovery, but its relationship with LEDVT has not been adequately explored. The Prognostic Nutritional Index (PNI) is a straightforward marker that includes serum albumin and lymphocyte count and serves as an indicator of both nutritional and immune status.

**Purpose:**

This research was done to examine the relationship between the PNI and LEDVT risk.

**Methods:**

This retrospective cohort study included 600 adult patients who underwent lower-limb duplex ultrasonography between January 2020 and December 2025. Clinical, demographic and laboratory data were gathered. PNI was calculated as: PNI = (10 × serum albumin [g/dL]) + (0.005 × total lymphocyte count [/mm^3^]). Patients have been divided into tertiles according to PNI. Logistic regression analyses were conducted to identify predictors of LEDVT, and the predictive performance of PNI was assessed using ROC curve analysis.

**Results:**

A total of 600 patients were studied, of whom 182 (30.3%) had LEDVT. The PNI of patients with LEDVT was significantly lower (41.1 ± 6.3) than that of patients without (48.2 ± 7.1, *p* < 0.001). The incidence of LEDVT declined steadily with increasing PNI tertiles (48.5, 29.0, and 13.5 in the low, moderate, and high PNI tertiles, respectively; *p*-trend <0.001). Lower PNI remained independently associated with LEDVT (adjusted OR 0.91/unit increase; 95% CI 0.88–0.94, *p* = 0.001) in multivariate analysis, alongside malignancy and immobilisation. Analysis of ROC curves revealed fair discriminative ability of PNI (AUC 0.74, 95% CI 0.70–0.78).

**Conclusion:**

There is a positive association between lower nutritional status (PNI) and the risk of LEDVT in hospitalised patients. PNI may serve as an easy, cost-effective biomarker for LEDVT risk stratification.

## 1 Introduction

Deep vein thrombosis (DVT) is a prevalent and potentially deadly disorder in which a thrombus forms in the deep veins, most often in the lower limbs. It is one of the causes of cardiovascular morbidity and mortality in the world; together with pulmonary embolism, it constitutes venous thromboembolism (VTE). Of clinical interest is lower extremity deep venous thrombosis (LEDVT), which is highly prevalent amongst patients admitted to a hospital and has been linked to both acute and long-term outcomes. Post-thrombotic syndrome (PTS) that occurs in a large percentage of patients may cause long-term pain, oedema, and venous ulcer, greatly affecting quality of life (1, 2).

A combination of endothelial injury, venous stasis, and hypercoagulability stems from a complex interaction that develops LEDVT. In addition to these classical processes, emerging evidence highlights the importance of systemic inflammation and nutritional status in the regulation of thrombotic risk. Inflammatory responses also contribute to endothelial dysfunction, activation of coagulation cascades, and inhibition of fibrinolysis and thrombus formation. Meanwhile, malnutrition (which is commonly manifested as hypoalbuminemia and impaired immune response) can further exacerbate this prothrombotic state by undermining vascular integrity and altering hemodynamic balance (3). Such results highlight the need to consider both inflammatory and nutritional risk factors when stratifying risk.

A composite measure is the prognostic nutritional index (PNI), which is based on serum albumin and peripheral lymphocyte count and indicates nutritional and immunological status. Originally designed as a tool to assess perioperative risk, PNI has since become a well-known tool in predicting prognosis in a broad spectrum of clinical conditions, such as malignancies, cardiovascular diseases, and infectious diseases (4). The low level of PNI has long been associated with systemic inflammation, immunosuppression, and worse patient outcomes, suggesting its potential as a combined biomarker of patient susceptibility.

Recent literature has examined the properties of PNI and thromboembolic diseases, with findings that lower PNI levels correlate with higher risk and poorer prognosis in conditions such as pulmonary embolism and cerebral venous sinus thrombosis (5, 6). These results validate the hypothesis that poor nutritional and immune status may contribute to thrombus formation and progression. Despite these findings, the association between PNI and LEDVT remains unclear. The majority of available risk assessment models for LEDVT focus on clinical, surgical, and immobilisation-related variables, with little attention to nutritional status as a potentially modifiable factor. Thus, the study hypothesised that the prognostic nutritional index (PNI) has an independent association with the occurrence of lower-extremity deep venous thrombosis (LEDVT) and assessed its predictive capabilities in hospitalised patients.

## 2 Methodology

### 2.1 Study design and setting

This research has been approved by the Ethics Committee of the Second Affiliated Hospital of Harbin Medical University, No. YLXJS2024-019. This retrospective observational study was conducted at a tertiary teaching hospital to assess the relationship between nutritional status, measured by the Prognostic Nutritional Index (PNI), and the incidence of lower-extremity deep venous thrombosis (LEDVT). Electronic medical records were used to screen hospitalised adult patients admitted between January 2020 and December 2025. A retrospective design was used to conduct a thorough examination of routinely collected clinical and laboratory data in a real hospital setting.

### 2.2 Study population

**Figure 1:**
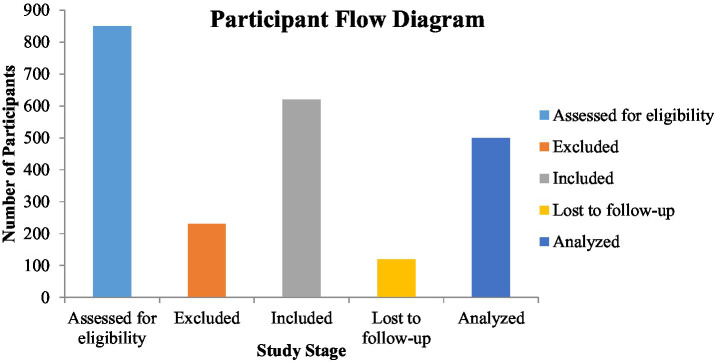
Flow diagram illustrating patient selection and exclusion criteria (*n* = 600).

The eligible patients were adult patients who were aged 18 years and above and who had been hospitalised because of clinical suspicion of venous thromboembolism and underwent lower limb duplex ultrasonography. Trained radiologists diagnosed LEDVT according to the established ultrasonographic criteria (reduced or absent venous compressibility, visualisation of intraluminal thrombus and impaired venous flow signal) (2). Those who had a history of previous venous thromboembolism, acute infectious/inflammatory conditions at hospital admission, chronic liver disease, nephrotic syndrome or haematological malignancies, as well as patients who had immunosuppressive therapy or albumin infusion, were excluded. Also, patients with incomplete clinical or laboratory data were not included. After implementing these criteria, 600 patients were included in the final analysis, out of 812 patients (Figure 1).

Thus, the study population is a selected sample of hospitalised patients undergoing ultrasonography to identify possible venous thromboembolism (excluding those with chronic liver disease, nephrotic syndrome, or haematological malignancies). Consequently, the results are limited to this particular group of clinical patients and may not be generalised to broader patient groups.

### 2.3 Data collection

The electronic medical records stored demographic and clinical data, including age, sex, body mass index (BMI), and key comorbidities, such as diabetes mellitus and hypertension. Clinical variables associated with increased thrombotic risk, such as the presence of malignancy, recent surgery within 30 days, and the duration of immobilisation during hospitalisation, were also documented. Laboratory values were measured within 24 h of hospitalisation. They included serum albumin level, total lymphocyte count, and C-reactive protein (CRP), which were consistent and showed reduced variation due to treatment.

The electronic records were not always available for data on prophylactic anticoagulant use, detailed functional status (e.g., frailty indices), and a comprehensive nutritional assessment beyond PNI, so they were not incorporated into the analysis.

### 2.4 Assessment of nutritional status

The Prognostic Nutritional Index was used to measure nutritional status, as it is a validated composite indicator of nutritional and immunological status. PNI obtained was determined by the following formula as originally described by (7).

$$\begin{array}{l} \mathrm{PNI}=(10\times \text{serum albumin}\;[g/\mathrm{dL}])\\ +(0.005\times \text{total lymphocyte count}\;[/{\mathrm{mm}}^{3}]) \end{array}$$

The patients were divided into low, moderate and high nutritional status based on the distribution of PNI values in the study population. Such stratification allowed evaluation of dose–response associations between nutritional status and the risk of LEDVT.

PNI was categorised into tertiles, and as a continuous variable to examine possible dose–response relationships. In contrast, tertile-based categorisation is not constructed based on accepted clinical thresholds, the receiver operating characteristic (ROC) curve analysis was conducted to determine a potential optimal cut-off value to be used clinically.

### 2.5 Outcome definition

The presence of lower-extremity deep venous thrombosis, established by duplex ultrasonography, was the main outcome of interest. The review of all imaging studies was performed by trained radiologists who were not informed of laboratory findings, thereby minimising potential diagnostic bias.

### 2.6 Statistical analysis

Statistical data were collected through SPSS Version 26.0. Continuous variables were evaluated for normality and presented as mean ± standard deviation or median with interquartile range, depending on the data, and categorical variables were presented as frequencies and percentages. Proper parametric or non-parametric tests were used to compare the patients with and without LEDVT. Logistic regression analyses have been used to investigate the relationship between PNI and LEDVT. Multivariate logistic regression models were used to identify independent predictors from variables that showed associations in the univariate analysis. Predictive performance of PNI was determined by means of receiver operating characteristic (ROC) curve analysis. A two-sided *p*-value less than 0.05 was regarded as significant. Given the retrospective design of the study, all findings are interpreted as associations rather than causal relationships.

## 3 Results

### 3.1 Patient characteristics

Due to the retrospective observational design, causality cannot be inferred from the observed associations. The findings should therefore be interpreted as hypothesis-generating rather than confirmatory. Of the 600 patients included in the analysis, 182 (30.3%) were diagnosed with LEDVT. The LEDVT group patients were also much older and had a slightly higher BMI than patients without thrombosis. The incidence of diabetes mellitus, hypertension, malignancy, protracted immobilisation, and recent surgery was significantly greater in patients whose LEDVT, which suggests that thrombotic risk factors are more pronounced in the topic population (Table 1).

**Table 1:** Baseline demographic and clinical characteristics of the study population.

Variable	Total (*n* = 600)	LEDVT (*n* = 182)	Non-LEDVT (*n* = 418)	*p*-value
Age (years)	56.3 ± 12.1	61.5 ± 11.0	53.9 ± 12.0	<0.001
Male sex, *n* (%)	328 (54.7%)	109 (59.9%)	219 (52.4%)	0.08
Body mass index (kg/m^2^)	26.9 ± 4.7	27.6 ± 4.9	26.6 ± 4.6	0.04
Diabetes mellitus, *n* (%)	201 (33.5%)	82 (45.1%)	119 (28.5%)	<0.001
Hypertension, *n* (%)	267 (44.5%)	96 (52.7%)	171 (40.9%)	0.007
Malignancy, *n* (%)	137 (22.8%)	71 (39.0%)	66 (15.8%)	<0.001
Immobilisation ≥3 days, *n* (%)	246 (41.0%)	109 (59.9%)	137 (32.8%)	<0.001
Recent surgery (≤30 days), *n* (%)	176 (29.3%)	74 (40.7%)	102 (24.4%)	<0.001

The values are displayed as either a number (%) or the mean ± standard deviation. The Chi-square test was used to compare categorical variables, whereas the Student’s *t*-test was used to evaluate continuous variables.

### 3.2 Laboratory values and nutritional status

The laboratory parameters between the two groups differed, as shown in Table 2. LEDVT patients had significantly lower serum albumin levels and reduced lymphocyte counts, resulting in a significantly lower PNI compared with patients without thrombosis. Moreover, the LEDVT group had significantly higher CRP levels, which indicate a higher inflammatory load. These results indicate that malnutrition, immunosuppression, and inflammation coexist in patients who present to LEDVT.

**Table 2:** Laboratory parameters and nutritional indices in patients with and without LEDVT.

Parameter	LEDVT (*n* = 182)	Non-LEDVT (*n* = 418)	*p*-value
Serum albumin (g/dL)	3.19 ± 0.45	3.71 ± 0.50	<0.001
Lymphocyte count (/mm^3^)	1,180 (860–1,520)	1,690 (1,340–2,050)	<0.001
C-reactive protein (mg/L)	17.6 (9.1–34.2)	8.1 (3.9–16.4)	<0.001
Prognostic Nutritional Index (PNI)	41.1 ± 6.3	48.2 ± 7.1	<0.001

Values are presented as mean ± standard deviation or median (interquartile range). Comparisons were performed using Student’s *t*-test or Mann–Whitney *U* test as appropriate. PNI was calculated as: (10 × serum albumin [g/dL]) + (0.005 × lymphocyte count [/mm^3^]).

### 3.3 Relationship between PNI tertiles and LEDVT

A comparison of LEDVT incidence across PNI tertiles indicated significant negative associations with thrombotic risk and nutritional status. Almost 50 % of the patients in the lowest tertile of PNI developed LEDVT, but the incidence decreased gradually in moderate and high tertiles of PNI. This linear-like trend was statistically significant and indicated a dose-dependent association with improved nutritional status on LEDVT. The correlation between PNI Tertiles and LEDVT is presented in Table 3.

**Table 3:** Incidence of LEDVT according to prognostic nutritional index tertiles.

PNI tertile	PNI range	Total (*n*)	LEDVT, *n* (%)	*p* for trend
Tertile 1 (Low)	≤42.0	200	97 (48.5)	
Tertile 2 (Moderate)	42.1–49.0	200	58 (29.0)	<0.001
Tertile 3 (High)	≥49.1	200	27 (13.5)	

PNI tertiles were defined based on the distribution of PNI values in the study population. The p for trend was calculated using the Chi-square test for trend. LEDVT incidence decreased progressively with increasing PNI.

### 3.4 PredictForms of LEDVT

Univariate logistic regression analysis revealed that age>60 years, diabetes mellitus, malignancy, immobilisation, high CRP levels, and low PNI were significant predictors of LEDVT. PNI was independently associated with LEDVT in a multivariate analysis that accounted for potential confounding factors. Every one-unit increase in PNI was associated with a 9% lower odds of LEDVT. Malignancy and long-term immobilisation were also strong independent risk factors, which is consistent with their established association with thrombotic risk (Table 4).

**Table 4:** Univariate logistic regression analysis for predictors of LEDVT.

Variable	Odds ratio (OR)	95% confidence interval	*p*-value
Age (per year increase)	1.04	1.03–1.06	<0.001
Diabetes mellitus	2.04	1.44–2.89	<0.001
Malignancy	3.43	2.32–5.07	<0.001
Immobilisation ≥3 days	3.05	2.13–4.38	<0.001
C-reactive protein (per mg/L)	1.03	1.02–1.05	<0.001
PNI (per unit increase)	0.89	0.86–0.92	<0.001

Univariate logistic regression was performed to assess associations between individual variables and LEDVT. ORs are presented per unit increase for continuous variables.

### 3.5 Diagnostic performance of PNI

**Figure 2:**
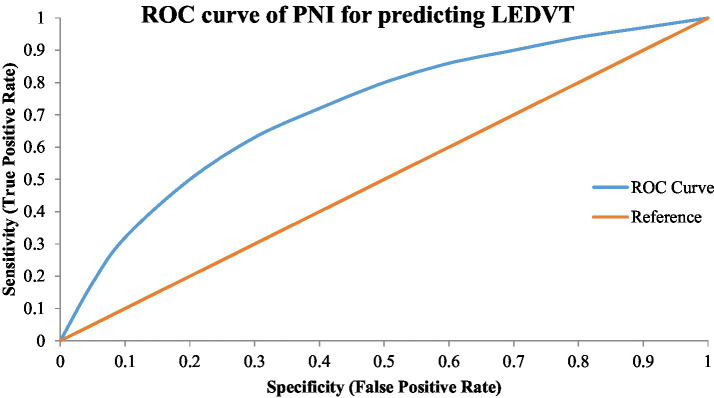
Receiver operating characteristic (ROC) curve of the prognostic nutritional index for predicting lower extremity deep venous thrombosis (LEDVT).

The analysis of receiver operating characteristic curves showed that PNI had fair discriminative ability for LEDVT (Table 5). The spot below the curve was 0.74 (95% CI: 0.70–0.78). The analysis of ROC has revealed that the optimal PNI cut-off value was 44.5, which offered a clinically relevant PNI cut-off value to stratify risks, an optimum sensitivity (72%) versus specificity (68%) ratio (Figure 2), which was in favour of the possibility of PNI being used as a simple and cost-effective method of identifying those patients who were at greater risk of LEDVT.

**Table 5:** Multivariable logistic regression analysis identifying independent predictors of LEDVT.

Variable	Adjusted OR	95% confidence interval	*p*-value
Age	1.03	1.01–1.05	0.001
Diabetes mellitus	1.61	1.09–2.38	0.02
Malignancy	2.52	1.63–3.91	<0.001
Immobilization ≥3 days	2.18	1.45–3.27	<0.001
C-reactive protein	1.02	1.00–1.04	0.03
Prognostic nutritional index (PNI)	0.91	0.88–0.94	<0.001

Multivariable logistic regression analysis was adjusted for age, sex, BMI, diabetes mellitus, hypertension, malignancy, immobilisation, recent surgery, and CRP. Adjusted ORs with 95% confidence intervals are shown. PNI remained independently associated with lower odds of LEDVT.

## 4 Discussion

The current research has shown that a reduced Prognostic Nutritional Index (PNI) is significantly associated with the occurrence of lower extremity deep venous thrombosis (LEDVT) in hospitalised patients. This association was also significant after correction for known clinical and inflammatory risk factors, and PNI demonstrated reasonable discriminative capacity in ROC analysis (AUC 0.74), implying that it is a useful adjunctive, but not diagnostic, marker. These results are consistent with prior research indicating an association between decreased PNI and thromboembolic risk (4, 6).

The patients in the study who had LEDVT were elderly and had more comorbidities, such as diabetes mellitus, hypertension, malignancy, and chronic immobility, which are known risk factors of thrombosis. Moreover, there were significant differences in laboratory results: serum albumin was lower, lymphocyte count was lower, and C-reactive protein concentration was higher in the LEDVT group. The results are consistent with the existing literature that shows that nutritional impairment and systemic inflammation are often comorbid in patients with venous thromboembolism (3, 5, 8).

There was an evident gradient across PNI tertiles, with a lower incidence of LEDVT in higher PNI tertiles, indicating a dose–response relationship between nutritional-immune status and thrombotic risk. Additionally, multivariate analysis showed that PNI remained significantly associated with LEDVT after adjustment for confounding variables. The results are in line with previous studies. Nakanishi et al. found that the pathogenesis of deep vein thrombosis in frail older adults was linked to impaired nutritional and functional status and to the role of diminished physiological reserves (9). Likewise, Sim et al. have shown that low PNI levels are associated with adverse outcomes after surgery (10). Mureșan et al. (8) also reported an association between nutritional-inflammatory indices, such as PNI, and thrombotic events and mortality, demonstrating a link between systemic nutritional status and thromboembolic disease.

The relationship between lower PNI and LEDVT could result from an interplay amongst nutritional status, immune function, and inflammation. The levels of serum albumin and lymphocytes reflect nutritional reserve and immune competence, and their decrease can indicate a more physiologically vulnerable state. Increased levels of C-reactive protein in patients with LEDVT are another indication of systemic inflammation, which is linked to endothelial dysfunction and activation of the coagulation pathway in venous thromboembolism (11). Clinically, PNI could be regarded as a complementary indicator for detecting patients at increased risk of thrombosis when used in combination with known clinical variables and laboratory indicators. Nonetheless, considering its average levels of discrimination, it cannot be applied to clinical decision-making on its own.

There are several limitations associated with this study. The retrospective design does not allow causal inferences, and the sample population comprised hospitalised patients with ultrasonographic findings suggestive of possible venous thromboembolism, which may limit generalizability. Moreover, the patient subset (chronic liver disease, nephrotic syndrome, and haematological malignancies) will be excluded, which can lead to selection bias and limit external validity. Unavailable variables (e.g., anticoagulant prophylaxis, functional status, longitudinal changes in nutritional or inflammatory markers) could also result in residual confounding. These considerations align with general limitations reported in the literature on venous thromboembolism and on biomarker-based risk assessment (12, 13). Thus, more prospective, multicenter investigations should be conducted to confirm these results in larger groups.

### 4.1 Strengths of the study

The study has the advantage of a large sample size (*n* = 600), which provides strong statistical power and reliable results. The use of real-life hospitalised patients increases the validity of external results and their extrapolation to different clinical environments. These findings suggest potential clinical relevance of using PNI as a simple, cost-efficient, and readily available laboratory marker, without the need for complex assessment methods. The exclusion of many confounders, such as age, comorbidities, inflammation, immobilisation, and malignancy, in the multivariable analysis also increases the study’s internal validity. The PNI tertile stratification allowed the assessment of a dose–response pattern, further supporting the fact that the positive effect of higher nutritional status is independent.

### 4.2 Limitation

There are several limitations in this study. First, the adopted retrospective observational design cannot establish a causal relationship; the results can only be interpreted as associations, not causality. Moreover, the targeted population of the study was patients admitted to hospitals and undergoing lower extremity duplex ultrasonography due to clinical suspicion of venous thromboembolism. This introduces selection bias, as the sampled patients are a higher-risk group than the general population, which may limit external validity and lead to an overestimation of the strength of the observed association.

Second, despite the validity and practicality of the Prognostic Nutritional Index (PNI) as a measure of nutritional and immune status, the index does not provide a full evaluation of nutritional status. Nutritional misclassification thus exists, since PNI fails to account for variables related to dietary intake, muscle mass, or comprehensive anthropometric and functional nutritional measurements. Such a constraint might have resulted in residual heterogeneity in the classification of nutritional status.

Third, some potential confounders were not available in the dataset, although some important clinical and inflammatory variables, including age, comorbidities, malignancy, immobilisation, and C-reactive protein, were adjusted. These include prophylactic use of anticoagulants, descriptive functional status (frailty), and longitudinal changes in nutritional or inflammatory markers. These variables may be missing, which could have affected the observed associations.

### 4.3 Future recommendations

Future research ought to be prospective and metacentric to confirm these findings on large groups of people. Future studies should explore whether nutritional status or PNI-guided strategies may be associated with changes in LEDVT risk. Serial PNI measurements could be considered at the time of hospitalisation and could help reveal the time dependence between nutritional status and thrombotic risk. A combination of PNI and other biomarkers, such as D-dimer or inflammatory indices, may improve predictive power and inform individualised thrombo-prophylaxis interventions in high-risk patients.

## 5 Conclusion

This paper shows that the presence of lower extremity deep venous thrombosis (LEDVT) amongst hospitalised patients was independently associated with Lower Prognostic Nutritional Index (PNI). This observation implies that the nutritional immune status can be useful for stratifying thrombotic risk. Although it may be a convenient and easy-to-use adjunctive measure for identifying patients at risk of increased exposure to LEDVT, its use in clinical practice must be undertaken with care. Future studies should be prospective, multicenter research to confirm these results and further elucidate their predictive power across different patient groups.

## Data Availability

The raw data supporting the conclusions of this article will be made available by the authors, without undue reservation.
